# Stereolithographic 3D Printing-Based Hierarchically Cellular Lattices for High-Performance Quasi-Solid Supercapacitor

**DOI:** 10.1007/s40820-019-0280-2

**Published:** 2019-06-01

**Authors:** Jianzhe Xue, Libo Gao, Xinkang Hu, Ke Cao, Wenzhao Zhou, Weidong Wang, Yang Lu

**Affiliations:** 1 School of Telecommunications Engineering, Xidian University, Xian, 710071 People’s Republic of China; 2 School of Mechano-Electronic Engineering, Xidian University, Xian, 710071 People’s Republic of China; 3 CityU-Xidian Joint Laboratory of Micro/Nano-Manufacturing, Shenzhen, 518057 People’s Republic of China; 4 Department of Mechanical Engineering, City University of Hong Kong, Kowloon, 999077 Hong Kong, SAR People’s Republic of China; 5 Nano-Manufacturing Laboratory (NML), Shenzhen Research Institute of City University of Hong Kong, Shenzhen, 518057 People’s Republic of China

**Keywords:** 3D printing, Lattices, Graphene, Supercapacitor, Porous structure, Stereolithography

## Abstract

3D printing-based supercapacitors have been extensively explored, yet the rigid rheological requirement for corresponding ink preparation significantly limits the manufacturing of true 3D architecture in achieving superior energy storage. We proposed the stereolithographic technique to fabricate the metallic composite lattices with octet-truss arrangement by using electroless plating and engineering the 3D hierarchically porous graphene onto the scaffolds to build the hierarchically cellular lattices in quasi-solid supercapacitor application. The supercapacitor device that is composed of composite lattices span several pore size orders from nm to mm holds promising behavior on the areal capacitance (57.75 mF cm^−2^), rate capability (70% retention, 2–40 mA cm^−2^), and long lifespan (96% after 5000 cycles), as well as superior energy density of 0.008 mWh cm^−2^, which are comparable to the state-of-the-art carbon-based supercapacitor. By synergistically combining this facile stereolithographic 3D printing technology with the hierarchically porous graphene architecture, we provide a novel route of manufacturing energy storage device as well as new insight into building other high-performance functional electronics.

**Electronic supplementary material:**

The online version of this article (10.1007/s40820-019-0280-2) contains supplementary material, which is available to authorized users.

## Highlights

- Stereolithographic 3D printing was introduced to fabricate polymeric lattices.
- Electroless plating was employed to make the lattices conductive and mechanically robust.
- Hydrogen bubbles were served as template for engineering the hierarchically porous graphene.
- The supercapacitor device holds great promise in the rational coupling of energy and power density.

**Electronic supplementary material:**

The online version of this article (10.1007/s40820-019-0280-2) contains supplementary material, which is available to authorized users.

## Graphical abstract

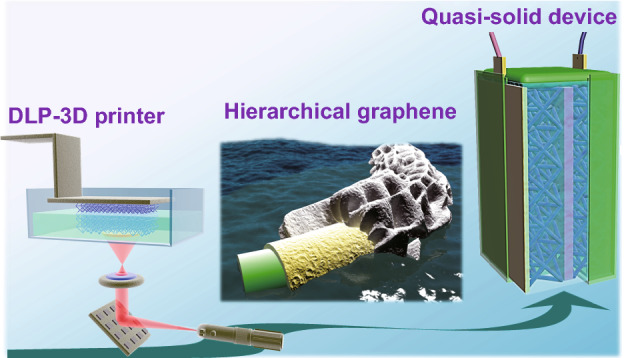

## Introduction

As potentially promising power candidate to cater for the rapid improvement in the wearable electronics, portable sensors, triboelectric nanogenerators, and solar cell, supercapacitors with a strong coupling of the energy and power density are highly demanded to meet its practical capability [1–10]. Building ordered 3D architecture with hierarchical pores is one of the most efficient ways to significantly improve related electrochemical performance for the energy storage, as the spatial configuration of the orderly porous structures can not only shorten the diffusion path of ions penetrating into the electrode materials but also enhance the robust mechanically structural integrity of electrode materials during long-time cycling compared to the conventional stochastic arrangements [11–17]. The free space within the 3D porous architecture can further serve as a buffer reservoir to allow enough electrolyte infiltration through the continuous channels, bringing high ion transportation. Thusly, self-assembly of using sacrificial templating and additive manufacturing known as 3D printing is generally two typical strategies used to prepare the orderly arranged 3D porous lattices [18, 19]. However, either the high cost for large-scale production or the limited custom-built geometric topology of the templating method restricts the practical application of building supercapacitor. Alternatively, 3D printing technology recently has been demonstrated superior structural manipulation and complex prototype abilities on energy storage and engineering application, which were extensively evidenced by our or other studies [19–27].

The direct ink writing (DIW)-based 3D printing technology so far was broadly employed for supercapacitor application because of its low-cost, adjustable, and scalable merits [28–30]. However, its rigid rheological requirement for the preparation of ink materials significantly limits the manufacturing of true 3D lattices in achieving improved energy storage ability. In this contest, many researches recently focused on stereolithography-based 3D printing due to its advantageous low cost, high efficiency, and ease of processability for fabricating almost any complex polymer-based structure [31, 32]. Yet to make the polymeric substrate materials conductive is a challenge which principally determines the fast electronic transportation in the supercapacitor. Carbonization or doping conductive nanoparticles into the photopolymer resin is practically limited because it not only weakens the mechanical integrality of the lattices but also suppresses the high-resolution ability of the stereolithography [31, 32]. Additionally, it is vital to further enhance the capacitive output of the lattices through modifying new materials such as the 2D graphene materials with high electrochemical performance [33]. To fully utilize the macroscopic electrochemical functions of graphene materials with high surface area, a promising way is to prepare large-scale assembly of graphene-based building blocks and graft their inherent properties onto 3D structures. However, an issue to be addressed is the serious stacking of the graphene sheets that significantly lower the ion-accessible surface area, limit the size of the channels, and increase the electronic resistance during the process of macroscale assembly [34]. There is thereby an urgent need, but it is still a significant challenge to rationally build 3D porous graphene electrode materials on the stereolithographic 3D-printed lattices.

Herein, we proposed the stereolithographic 3D printing technology to fabricate the rationally designed stretching-dominated polymeric lattices with octet-truss topology as the basic substrate for supercapacitor. The electroless plating manner was then employed to make the substrate conductive and enhance mechanical behavior. To fabricate the hierarchically porous architecture into the reduced graphene oxide (rGO), we used the hydrogen bubbles as soft templates which were inspired by our earlier study [35]. Through these above-advanced routes, a hierarchically porous graphene-based composite lattice was successfully fabricated, and its great potential application in quasi-solid supercapacitor device with high energy density was carefully investigated and demonstrated. This design concept that combines the 3D printing with hierarchically porous graphene architecture would not only guide the researchers to further improve the energy storage for supercapacitors through a new way but also show a novel concept for engineering other functional electronics.

## Experimental Section

### Preparation of the Composite Lattices

Firstly, the rationally designed computer-aided design (CAD) file of the stretching-dominated octet-truss architecture was directly imported into the customized 3D software Creation Workshop with slicing distance of 25 µm for the 3D printing with an exposure time of 20 and 8 s for bottom and left parts of the sample, respectively. The 3D geometry structure of the polymeric lattices was chosen to be 5 unit cells wide by 5 unit cells long by 1 unit cell tall. The overall size of the fabricated lattices was 20.00 × 20.00 × 4.00 mm^3^, with a strut diameter of 500 µm. The polymer lattices were synthesized acrylate-based UV photosensitive resin (L101, Nova 3D), mainly composed of non-toxic acrylic polyester and curable using a 405-nm light. The sample was then carefully taken away from the substrate and immersed into the alcohol solution and deionized (DI) water with ultrasonic for 1 min, respectively, to sufficiently remove the uncured photopolymer. After drying it enough, the printed lattice prototype was electroless deposited with nickel–phosphorus (NiP) metal layer to make it conductive. This process is similar to our earlier reports [36]. Briefly, the dried sample was thoroughly immersed into 10 g L^−1^ of SnCl_2_ for 20 min, washed gently with DI water and further dried at room temperature. Upon drying, the treated polymeric lattices were again placed into an activation solution containing 0.25 g L^−1^ PbCl_2_ and 10 mL L^−1^ hydrochloric acid for 20 min. Similarly, after being washed with DI water and dried, the activated lattices were carefully immersed into the water bath and kept at 90 °C for 15 min to obtain the metallic composites. For the NiP plating solution, 40 g L^−1^ of NiSO_4_∙5H_2_O, 20 g L^−1^ of sodium citrate, 10 g L^−1^ of lactic acid, and 1 g L^−1^ of dimethylamine borane were mixed homogeneously with further dilution of fivefold. With regard to the copper plating solution, its composition is similar to the reports elsewhere with a slight modification [37]. Then, 14 g L^−1^ of CuSO_4_ 5H_2_O, 12 g L^−1^ of NaOH, 16 g L^−1^ of potassium sodium tartrate, 20 g L^−1^ of EDTA·2Na, 26 mL L^−1^ of HCHO, 20 mg L^−1^ of 2,2′-dipyridyl (− 1), and 10 mg L^−1^ of potassium ferrocyanide were accordingly prepared. Note that the prepared temperature is kept at 45 °C during the copper plating procedure. The final composite lattices were obtained after DI water washing and drying. More detailed information is found in supporting information.

### Preparation of the Porous rGO

The rGO layer acting as the electrode materials was synthesized via the electroplating way. To obtain the 3D porous rGO, the synthesized NiP-coated lattices, namely NiP/polymer that serves as the scaffold (or current collector), were directly immersed into the 3 mg mL^−1^ of GO solution (Hengqiu Tech. Inc., Suzhou) mixed with 0.1 mL L^−1^ HCl for electrodepositing active materials. After electrodeposited for 5 min under the voltage of 30 V (the Pt plate was regarded as the counter electrode), the as-synthesized porous rGO hydrogel (rGO-1) was washed with abundant DI water and further reduced with a 0.1 M ascorbic acid at 90 °C for 6 h (rGO-2) [38]. Finally, the as-synthesized sample was freeze-dried at − 80 °C for 24 h and dried in a vacuum oven and the mass loading of sample was to be ~ 2.2 mg cm^−2^.

### Preparation of the Quasi-Solid Supercapacitor

The gel-type electrolyte was prepared according to our previous procedure [6], or similar methods reported elsewhere [39]. Polyvinyl alcohol (PVA, 6 g) was dissolved in DI water (50 mL) under vigorous magnetic stirring at 100 °C until the solution became transparent enough, and 20 mL of KOH solution (0.3 g mL^−1^) was added slowly. The prepared rGO lattices were immersed into the above gel electrolyte for 10 min, and it was repeated three times. Then, two lattices were carefully compressed together with the commercial polypropylene (PP) membrane with a thickness of 0.15 mm as separator into the 3D-printed sealing shell. Finally, it was carefully sealed by the 3D-printed shell.

## Characterization

### Structural Characterization

The structural and morphological information was characterized by field emission scanning electron microscope (FESEM, Quanta 450, 20 kV) equipped with energy-dispersive X-ray (EDX) and the transmission electron microscope (TEM, JEM 2100F). The phase and crystal structures were identified by X-ray diffraction (XRD, Rigaku SmartLab, Cu Kα, 1.5418 Å). X-ray photoelectron spectroscopy (XPS) was conducted on VG ESCALAB 250 spectrometer with Al Kα X-ray radiation.

### Mechanical and Electrochemical Characterizations

The mechanical performance of the lattice was tested in Material Test System (Alliance RT 30kN, USA). At least three samples were tested to obtain reliable values.

The electrochemical tests were associated with the cyclic voltammetry (CV) and galvanostatic charge–discharge (GCD) curves as well as the electrochemical impedance spectroscopy (EIS) measurements. (Frequency ranged from 10^−1^ to 10^5^ Hz.)

The areal capacitance (C, F cm^−2^) of the electrode was calculated by Eq. 1:

1 $$C = \frac{i\Delta t}{S\Delta V}$$

where *i* (A) is the current, Δ*t* (s) is the discharge time, *S* is the surface area, and *V* (V) is the potential range.

Note that for the device testing, the surface area is the total device area. And thus, the energy density (*E*, Wh cm^−2^) and power density (*P*, W cm^−2^) are based on Eqs. 2 and 3, respectively.

2 $$E = \frac{1}{2} \times C \times \frac{{(\Delta V)^{2} }}{3600}$$

3 $$P = \frac{E}{t} \times 3600$$

where *C* (F cm^−2^) is the areal capacitance and Δ*V* (s) is the potential range.

## Results and Discussion

**Fig. 1:**
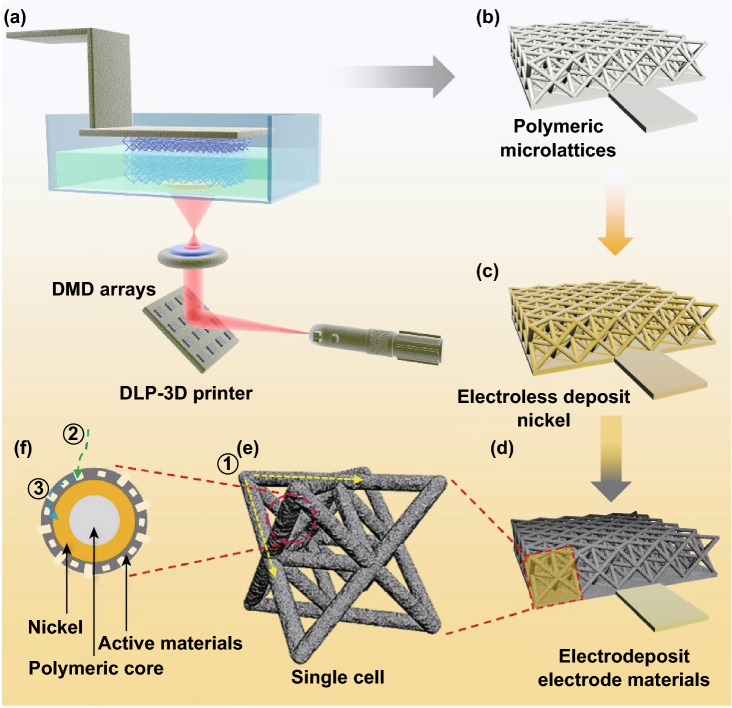
Schematic illustration of the procedure for fabricating the composite lattices. **a** The polymeric lattices fabricated by the DMD-based DLP 3D printer. **b** The as-synthesized polymeric lattice. **c** The NiP/polymer composite lattices fabricated using electroless plating. **d** The rGO electrode materials electrodeposited on the composite lattices structure. **e** The enlarged topology view of the single octet-truss structure. **f** The rationally fabricated rGO with a porous structure. The ① ② ③ designate the pathway of the ions and electron transportation

The design considerations of the lattice principally include two items closely related to its mechano-electrochemical performances before the practical manufacturing of the lattices by the 3D printing. On the one hand, the topological configuration determines the stretching-dominated or bending-dominated type of the cellular materials, which already has been discussed in either our or other studies [40, 41]. In this study, a typically known octet-truss architecture was rationally employed because of its relatively high specific strength corresponding to less constituted materials used [42]. On the other hand, the feature size of one strut should be small enough of ~ 500 μm (the most stable size that the 3D printer can reach) to increase the deposited specific surface area of the whole scaffold for electrode materials [11]. The procedure for manufacturing the composite lattices is shown in Fig. 1. In this study, digital light processing (DLP)-based 3D printer with a wavelength of 405 nm for the UV light is employed because of its fast printing speed based on the ability to solidify the entire polymeric layer once via the digital mirror device (DMD) arrays (Fig. 1a) [43]. The electroless deposition route is used to make polymeric lattices conductive with deposition of a thin NiP layer (Fig. 1b, c). Different with the conventional manner to directly deposit the dense electrode film on the substrate, we smartly electrodeposited the 3D porous rGO on the metallic strut to sufficiently use the periodic pores of the composite lattices and build aligned pores enabling fast ion transport to enhance electrochemical performance (Fig. 1d–f).

**Fig. 2:**
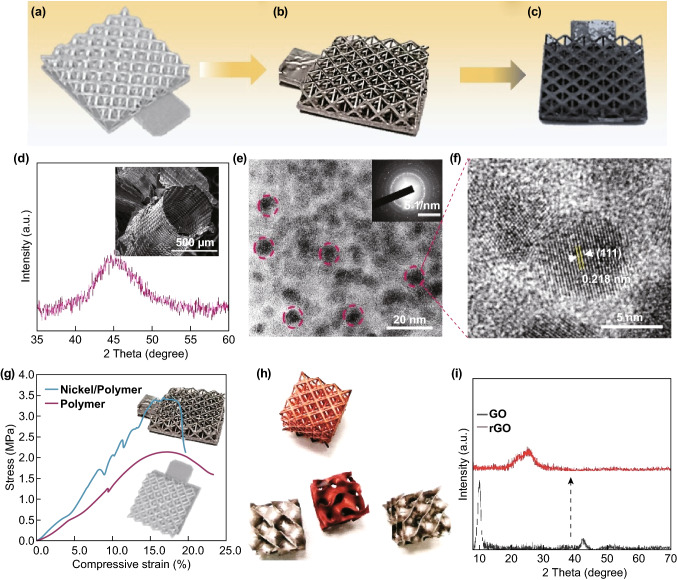
Structural characterization of the metallic composite lattices. Digital images of **a** polymeric lattices, **b** NiP/polymer lattices, **c** rGO composite lattices. **d** XRD of the hollow NiP lattices; inset is the corresponding FESEM images of single hollow NiP structure. **e** TEM and **f** HRTEM images of the NiP film. **g** Compression test of pure polymeric and metallic composite lattices. **h** Large-scale fabrication of the metallic (NiP, CuP) composite lattices. **i** XRD patterns of GO and the final rGO

The as-synthesized lattices are shown in Fig. 2. Well-designed and complex polymeric structure with unique texture produced during the stereolithography process was successfully fabricated (Figs. 2a, S2a). The NiP-coated lattices with shining color still retained its original structural integrity with the layer’s thickness of ~ 5 μm (Figs. 2b, S2). The 3D porous rGO was also successfully and uniformly deposited onto the metallic composite lattices (Fig. 2c), showing a good adhesion on the polymeric substrate. Besides, the rGO also shows excellent adhesion on other substrate such as smooth nickel wire, mesh and even plate (detailed information is found in Fig. S3), indicating its versatile and general merits to fabricate the rGO on various substrates. To obtain more structural information of the metallic film, we directly removed the polymer core by immersing the composite lattices in 6 M of NaOH solution at 60 °C and keeping for 24 h to get the hollow metallic lattices (as shown in the inset of Fig. 2d and the digital optical images of Fig. S4). The broad peak of the XRD for the metallic film centered around 40°–50° indicates the characteristic of the NiP alloy (Fig. 2d) [44, 45], which is further confirmed by the TEM and SAED pattern in Fig. 2e. The well-defined lattice fringes with a distance of 0.218 nm in the HRTEM images belong to the (111) plane of the hexagonal Ni_2_P (Fig. 2f), which is in good accordance with other reports and the SEM–EDS results in Fig. S5. Additionally, the Ni_2_P composite lattice exhibits high stress-bearing ability almost with no loss of compressive strain, demonstrating the mechanical enhancement and structural integrity after being coated with the Ni_2_P film (Fig. 2g). Moreover, this kind of fabrication was readily scalable as the procedure was conducted in a solution way. Figure 2h shows its manufacturing ability to fabricate other complex structure and even the Cu lattices. The metallic lattice showed superior conductivity of 2 Ω cm^−2^, indicating its potential in regard to the current collector and conductive scaffold. Finally, the XRD patterns showed that the featured peak of GO located at around 10° disappeared and a broad peak was observed at 24.5°, revealing the successful reduction of the GO on the Ni_2_P composite lattice (Fig. 2i) [46].

**Fig. 3:**
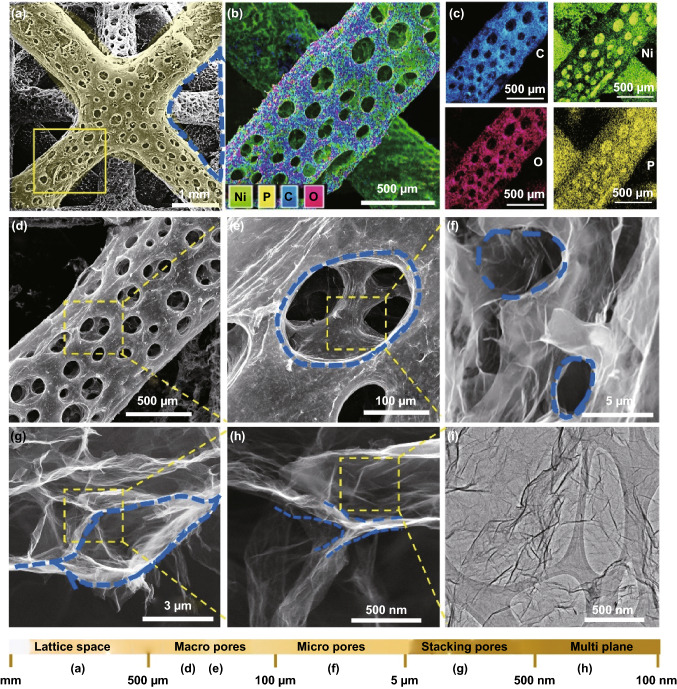
Hierarchically porous lattices structure. **a** FESEM image of the individual porous lattice. **b**–**d** SEM–EDX mapping and FESEM image of the single porous structure. **e** The enlarged view of the continuous porous structure. **f** The micropores existed on the side wall of the rGO. **g** The pores produced by the stacking by the graphene sheets. **h** The pores made from multilayers of the graphene. **i** TEM image of the graphene sheets with wrinkling and wave-like structure

To further observe the morphology of the rGO product, corresponding low- and high-magnification FESEM images are shown in Fig. 3. Figure 3a indicates the successful engineering of the porous structure on the metallic lattices. This unique, interesting, and hierarchically porous architecture allows effective utilization of the electrode volume and thus increases areal capacitance. Combining with this proposed 3D printing technology, the hierarchical porosity between the truss members within one lattice can be tuned so that the effective stress relief can be achieved [11]. The SEM–EDX results (Figs. 3b, c and S6) are in accordance with that of Fig. 2i. Each strut in lattices was made up of 1-mm-diameter rGO with a continuous pore size of ~ 200 μm (Fig. 3d, e), which is beneficial to the enhancement of the penetration of the electrolyte ions. Further, multi-cavities with diameter of 5 μm were found on the side wall of the rGO (Fig. 3f), and the stacking pores (Fig. 3g) are produced by the porous multi-plane graphene sheets (Fig. 3h). This special hierarchically porous structure and the wrinkling graphene sheet (Fig. 3i) are expected to improve the electrochemical performance based on more diffusion paths generated by the lattice and porous rGO. The formation mechanism of the porous architecture is similar to our previous study of fabricating the hollow and porous copper foam [35]. Specifically, at initial state, the GO and H^+^ (from hydrochloric acid solution) were continuously appealed to the substrate by the applied voltage. During this process, the GO would be partially reduced to rGO, while the H^+^ shall be reduced to H_2_ bubbles on the substrate. Due to the escape of the H_2_ bubbles, the hollow and porous structure would be fabricated (Fig. 3a). Besides, the ice crystals would also be served as the template for forming the frameworks in Fig. 3g in the posttreatment of freeze-drying.

**Fig. 4:**
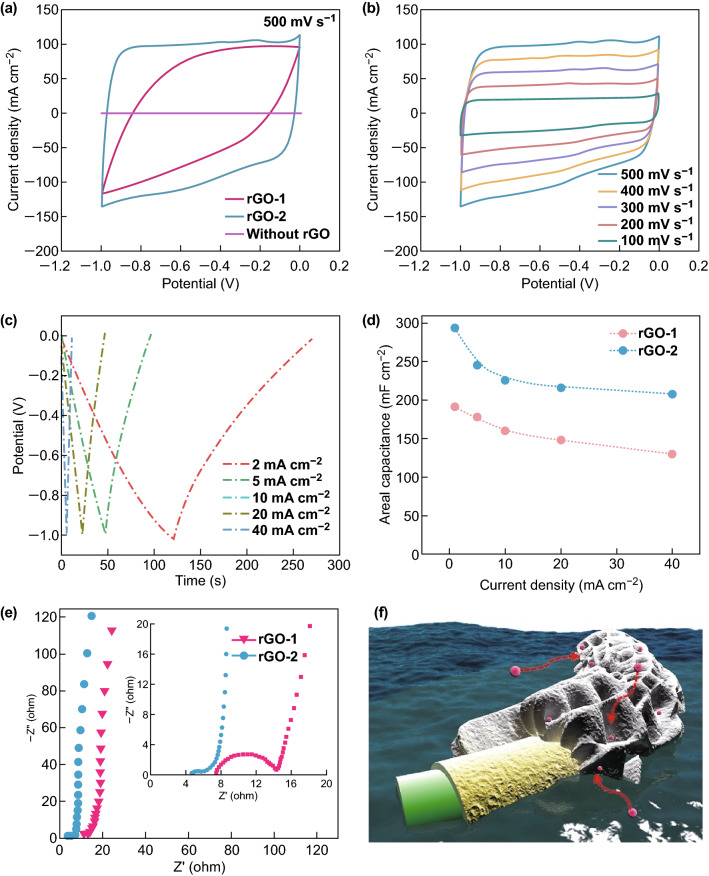
Three-electrode electrochemical performance of the rGO. **a** CV curves of various samples at a scan rate of 500 mV s^−1^. **b** CV curves of the rGO-2 at various scan rates from 100 to 500 mV s^−1^. **c** GCD curves of the rGO-2 at various current densities from 2 to 40 mA cm^−2^. **d** The corresponding areal capacitance of rGO-1 and rGO-2. **e** The EIS of rGO-1 and rGO-2. **f** The schematic illustration of the electrode in the process of charging and discharging

To examine its potential serving as an electrode in the energy storage device, we tested the electrochemical performance of the rGO on the application of supercapacitor in a three-electrode way. After further reduction by the ascorbic acid, the rGO-2 showed larger integral area and near rectangular shape compared to the rGO-1 or the bare metallic lattices at a scan rate of 500 mV s^−1^, indicating the successful preparation and enough reduction in the rGO-2 lattices (Fig. 4a and XPS analysis in Fig. S7), and the capacitance supplied from the NiP can almost be neglected. The CV curves even at a rate of as high as 500 mV s^−1^ remained no distortion owing to the hierarchically porous structure that provides fast ion transfer/diffusion pathway and rapid ion response (Fig. 4b). The typical behavior of the electrical double-layer capacitance (EDLC) was also confirmed by the GCD curves in Fig. 4c. More significantly, the rGO-2 showed large areal capacitance of 293.4 mF cm^−2^ at a current density of 2 mA cm^−2^ and still retained 208 mF cm^−2^ when the current density increased 20-fold, but the rGO-1 exhibited inferior performance of 191.6 mF cm^−2^ (Figs. 4d and S8), and this value is comparable to the recently reported 3D-printed graphene substrate (24 mF cm^−2^) for MnO_2_ loading [17]. To further ascertain the derivation of the superior electrochemical performance of the rGO-2, the EIS was employed. The Warburg-type lines demonstrated the fast ion transportation in the rGO-2 because of the shorter projected length on the real axis [47]. Small semicircle is mainly derived from the residual functional group of rGO. Further, the rGO-2 showed lower electrode resistance compared with the rGO-1 in the high-frequency region, indicating the better reduction by using the ascorbic acid (Fig. 4e). In conclusion, these results demonstrate the exceptional electrochemical performance is mainly derived from its fast ion diffusion that resulted from the hierarchically porous structure and improved conductivity owing to the better chemical reduction (Fig. 4f). Note that the plating time also is a significant effect on its capacitive performance as shown in Fig. S9.

**Fig. 5:**
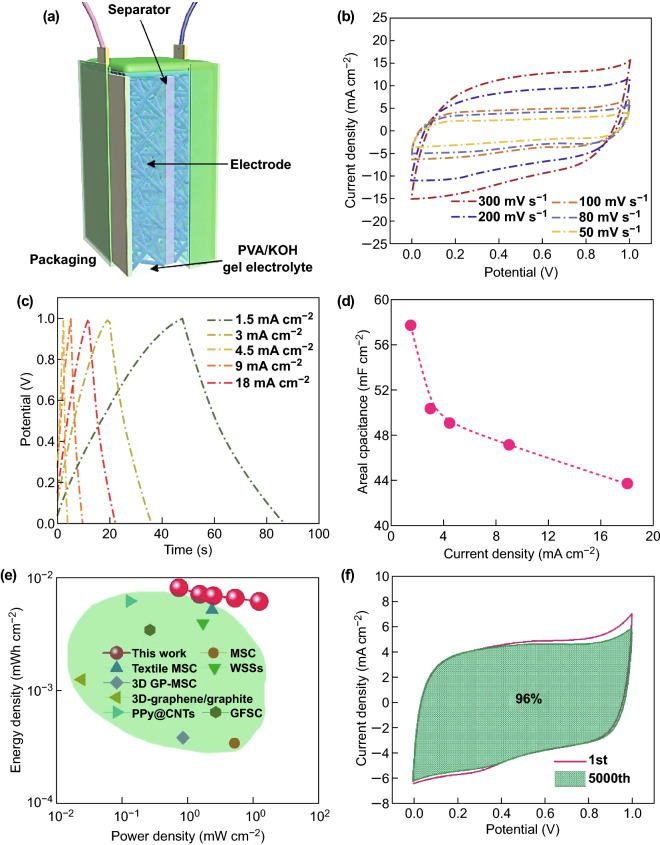
Electrochemical performance of the device. **a** Schematic illustration of the fabricated quasi-solid supercapacitor. **b** CV and **c** GCD curves of the device. **d** The specific capacitance of the device as a function of the current density. **e** Typical Ragone plots for the present symmetric system based on the full device, compared with other high-end supercapacitors. **f** The long cycling stability of the device

To prove the electrode in practical application, the device was assembled by two electrodes using the gel electrolyte and sealed with the 3D-printed packing box (Fig. 5a). The capacitive performance of the assembled device was investigated with CV curves and GCD curves. As shown in Fig. 5b, the device exhibited nearly similar shape without obvious distortion at various scan rates of from 50 to 300 mV s^−1^, indicating the assembled device has a fast electrochemical response. Figure 5c shows the GCD curves of the device at different applied current densities ranging from 1.5 to 18 mA cm^−2^. The specific capacitance can nearly reach 57.75 mF cm^−2^ (259.9 mF cm^−3^ and 13.1 F g^−1^) at current density of 1.5 mA cm^−2^ and till remained 43.7 mF cm^−2^ (196.7 mF cm^−3^) at an enlarged current density of 18 mA cm^−2^ in Fig. 5d. This value is superior to other reported carbon-based materials in solid-state carbon-based supercapacitor device such as 3D-GCAs (4.76 Fg^−1^) [16], B-3D-PCP MSC (2.95 mF cm^−2^) [32], FCSC (38.2 mF cm^−2^) [48], TiN@C (19.4 mF cm^−2^) [49], and porous graphene films (55.4 mF cm^−2^) [50]. The Ragone plot of the present system, which is a characteristic indication of both energy density and power density, is shown in Fig. 5e. The maximum energy density of 0.008 mWh cm^−2^ (0.036 mWh cm^−3^) is achieved with a power density of 0.75 mW cm^−2^ (3.4 mW cm^−3^). The maximum power density also reached 12.56 mW cm^−2^ (56.52 mW cm^−3^) at an energy density of 0.0061 mWh cm^−2^ (0.027 mWh cm^−3^). The energy density is significantly higher than most reported values to date for porous graphene-based materials, such as MSC (0.22 μwh cm^−2^, 0.37 mW cm^−2^) [51], textile MSC (0.51 μwh cm^−2^, 2.4 mW cm^−2^) [52], WSSs (3.9 μwh cm^−2^, 1.5 mW cm^−2^) [53], 3D GP-MSC (0.38 μwh cm^−2^, 0.86 mW cm^−2^) [54], 3D-graphene/graphite (1.24 μWh cm^−2^, 24.5 μW cm^−2^) [55], PPy@CNTs@urethane elastic fibers (6.13 µWh cm^−2^, 0.133 mW cm^−2^) [56], and GFSC (3.4 µW h cm^−2^, 0.27 mW cm^−2^) [57]. More importantly, our assembled device showed superior long cycling stability with 96% retention after 5000 cycles, demonstrating its great potential in practical application (Fig. 5f). With the encouraging results from this proof of concept evaluation, our future work will focus on demonstrating the electrochemical improvement in the downsized micro/nanolattices as a basic current collector and investigate how the geometric topology and electrode materials synergistically improve the energy and power densities.

## Conclusions

In summary, the hierarchically cellular lattices were rationally constructed by combining the stereolithographic 3D printing technology with the hierarchically porous graphene electrode materials. Thanks to the unique architecture and good intrinsic conductivity of the composite lattices, fast transport for electrons and ionic kinetics is well improved. Thusly, the as-synthesized supercapacitor device achieved superior areal capacitance (57.75 mF cm^−2^) and rate capability (70% retention, 2–40 mA cm^−2^) as well as long lifespan (96% after 5000 cycles), which are comparable to the state-of-the-art carbon-based supercapacitor device. Moreover, the admirable maximum energy density (0.008 mWh cm^−2^) and power density (12.56 mW cm^−2^) evidenced their future in practical application and the smart design concept. This study opens a new door in manufacturing the energy storage device in addition to the conventional DIW method and provides a novel concept in rationally building low tortuosity and fast transportation of ordered composite lattices through the synergetic combination of the 3D printing with self-assembly.

## Electronic supplementary material

Below is the link to the electronic supplementary material.

Supplementary material 1 (PDF 501 kb)
