# Supplementary material for: Stereolithographic 3D Printing-Based Hierarchically Cellular Lattices for High-Performance Quasi-Solid Supercapacitor
Source: Nanomicro Lett. 2019 Jun 1;11:46. doi: 10.1007/s40820-019-0280-2 (PMC7770913; doi:10.1007/s40820-019-0280-2)
Supplement: Supplementary file 1 — Supplementary material 1 (PDF 501 kb) [file 40820_2019_280_MOESM1_ESM.pdf]

Supporting Information for

Stereolithographic 3D Printing-Based Hierarchically Cellular Lattices for High-performance Quasi-Solid Supercapacitor

Jianzhe Xue[1], Libo Gao[2, 3, *], Xinkang Hu[2], Ke Cao[4], Wenzhao Zhou[3, 5], Weidong Wang[2, 3, *], Yang Lu[3, 4, 5, *]

[1]School of Telecommunications Engineering, Xidian University, Xian 710071, People's Republic of China

[2]School of Mechano-Electronic Engineering, Xidian University, Xian 710071, People's Republic of China

[3]CityU-Xidian Joint Laboratory of Micro/Nano-Manufacturing, Shenzhen 518057, People's Republic of China

[4]Department of Mechanical Engineering, City University of Hong Kong, Hong Kong SAR, Kowloon 999077, Hong Kong, People's Republic of China

[5]Nano-Manufacturing Laboratory (NML), Shenzhen Research Institute of City University of Hong Kong, Shenzhen 518057, People's Republic of China

Jianzhe Xue, Libo Gao, and Xinkang Hu contributed equally to this work

*Corresponding authors. E-mail: lbgao@xidian.edu.cn (Libo Gao); wangwd@mail.xidian.edu.cn (Weidong Wang); yanglu@cityu.edu.hk (Yang Lu)

S1 Process of Electroless Deposition

The electroless deposition mainly involves three steps of sensitization, activation and electroless deposition. Both the sensitization and activation are used to form an active catalytic layer, which is composed of Pd nucleus contributing to the reductive property of Sn^{2+} ions to Pd^{2+} ions, as shown in Eq. (S1):

$$Sn^{2+} + Pd^{2+} \rightarrow Sn^{4+} + Pd \qquad (S1)$$

Pd can then regard as a catalyzer in the process of the electroless Ni-P plating at initial deposition stage.

During the electroless deposition, the sustainable Ni-P deposition including the catalytic and autocatalytic process, in which the Pd produced above is homogeneously attached on the polymeric surface as catalysis in the replacement reaction. It mainly involves the following steps:

$$H_2PO_2^- + H_2O \xrightarrow{Pd/Heating} HPO_3^{2-} + 2H\cdot + H^+ \quad (S2)$$

$$Ni^{2+} + 2H\cdot \rightarrow Ni + 2H^+ \quad (S3)$$

$$H_2PO_2^- + 2H\cdot \rightarrow H_2O + OH^- + P \quad (S4)$$

$$H_2PO_2^- + H_2O \xrightarrow{Ni/Heating} HPO_3^{2-} + 2H\cdot + H^+ \quad (S5)$$

During this process, the $H_2PO_2^-$ ions reacts with H_2O to produce hydrogen free-radical with the existence of Pd under heating condition. Then, the Ni^{2+} and $H_2PO_2^-$ is reacted with nascent hydrogen to produce Ni and P, respectively. Therefore, the co-precipitation of Ni and P alloy will be produced on the 3D polymeric lattices. Due to the easy penetration of the deposition solution into the inner space of the lattices, therefor it is well coated with a Ni-P alloy.

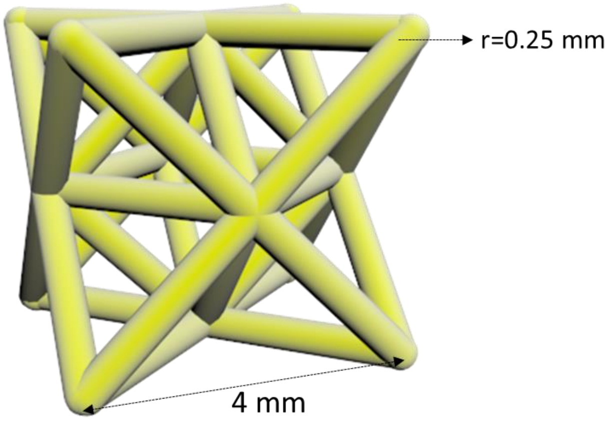

Fig. S1 Single unit of the cell structure

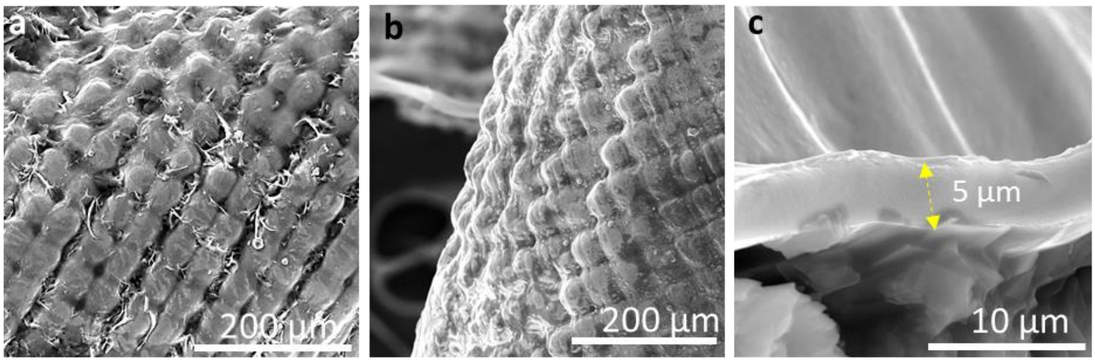

Fig. S2 Field emission scanning electron microscope (FESEM) images of **a** polymeric structure; **b** NiP/polymer lattice, and **c** pure NiP layer

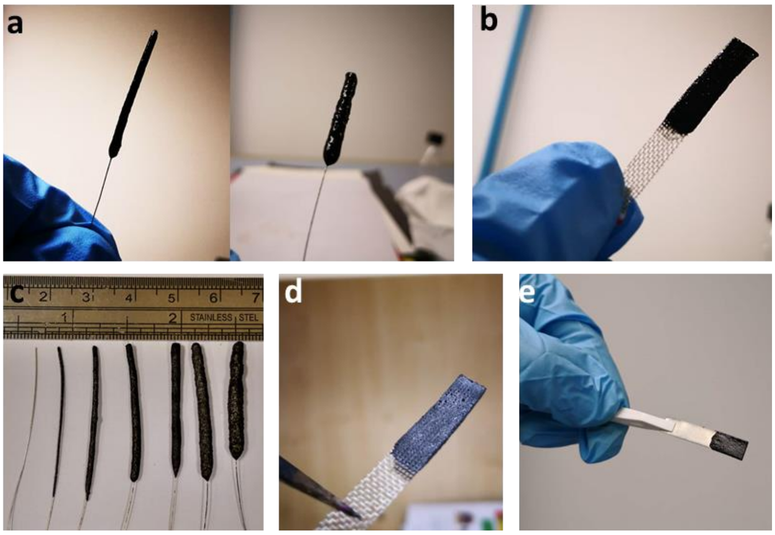

Fig. S3 Optical microscopy images of the rGO on **a** the smooth nickel wire **b** nickel mesh before freeze-drying. And the corresponding dried sample **c, d** without any behavior of peeling off. **e** Dried rGO on the smooth nickel plate

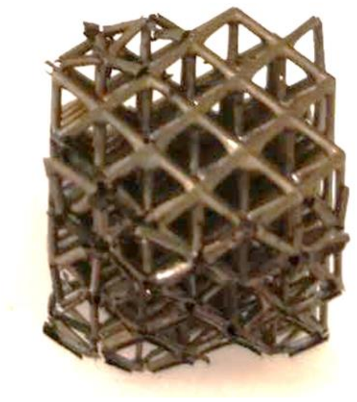

Fig. S4 Digital optical image of the hollow NiP lattices

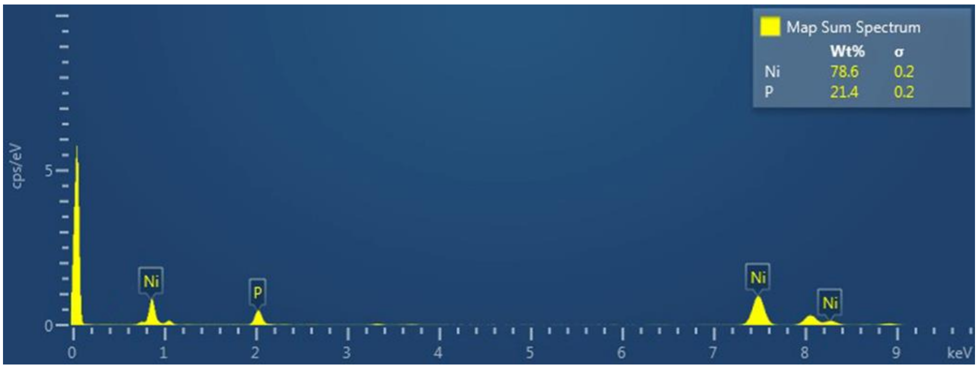

Fig. S5 SEM-EDX of the NiP lattices

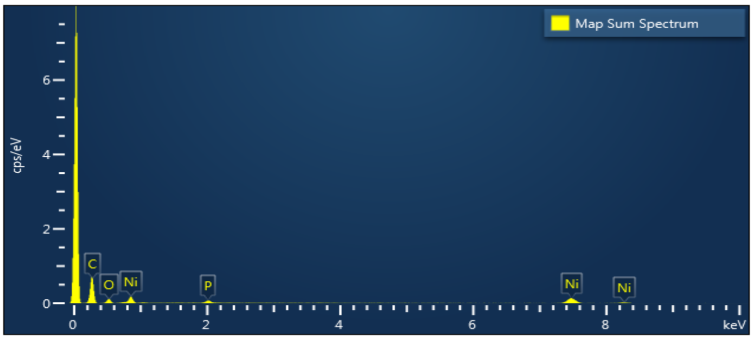

Fig. S6 SEM-EDX of the rGO composite lattices

The reduction of GO is examined by comparing the X-ray photoelectron spectroscopy (XPS) spectra of GO, rGO-1, and rGO-2. The two peaks centered at 286 and 533 eV are in accordance with the C and O elements, respectively [S1]. A C/O atomic ratio was calculated to be 2.1 for GO, while the ratio of C/O increases from 4.08 for rGO-1 to 7.3 for rGO-2, demonstrating that most of the oxygen-containing groups are removed and a better reduction for rGO-2.

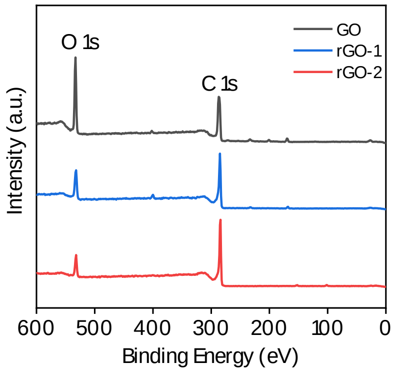

Fig. S7 XPS wide scan spectra of C 1s and O 1s

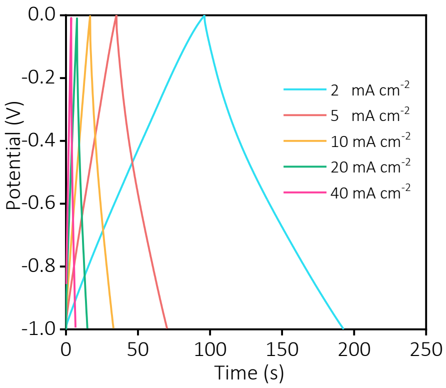

Fig. S8 GCD curves of the rGO-1 composite lattices at various current densities

From the CV curves of rGO-2 fabricated for different time, although increasing time would get the thick electrode materials, but the distorted behavior was observed due to the increased resistance and tortuous pathway for the ions and electrons. Therefore, we choose the rGO deposited for 5 min as the typical sample in this study.

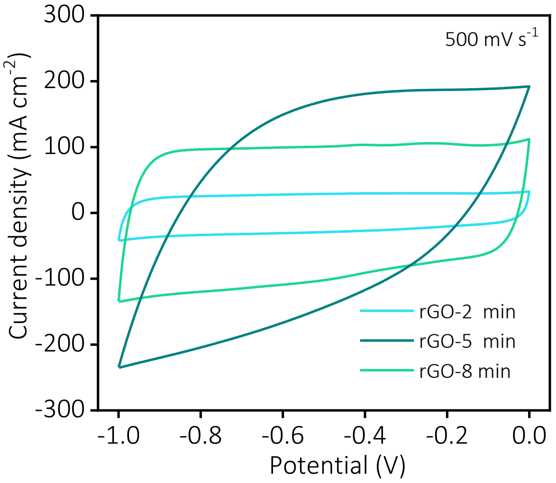

Fig. S9 CV curves of the rGO-2 fabricated for different time

Supplementary References

[S1] Z. Zhang, M. Liu, X. Tian, P. Xu, C. Fu, S. Wang, Y. Liu, Scalable fabrication of ultrathin free-standing graphene nanomesh fi lms for flexible ultrafast electrochemical capacitors with AC line-filtering performance. Nano Energy **50** 182–191 (2018). http://doi.org/10.1016/j.nanoen.2018.05.030
